# A Perspective on CRN Proteins in the Genomics Age: Evolution, Classification, Delivery and Function Revisited

**DOI:** 10.3389/fpls.2017.00099

**Published:** 2017-02-03

**Authors:** Tiago M. M. M. Amaro, Gaëtan J. A. Thilliez, Graham B. Motion, Edgar Huitema

**Affiliations:** ^1^Division of Plant Sciences, University of DundeeDundee, UK; ^2^Dundee Effector ConsortiumDundee, UK; ^3^Cell and Molecular Sciences, The James Hutton InstituteInvergowrie, UK

**Keywords:** oomycetes, *Phytophthora*, effectors, CRN, nucleus, immunity, CR-toxins

## Abstract

Plant associated microbes rely on secreted virulence factors (effectors) to modulate host immunity and ensure progressive infection. Amongst the secreted protein repertoires defined and studied in pathogens to date, the CRNs (for CRinkling and Necrosis) have emerged as one of only a few highly conserved protein families, spread across several kingdoms. CRN proteins were first identified in plant pathogenic oomycetes where they were found to be modular factors that are secreted and translocated inside host cells by means of a conserved N-terminal domain. Subsequent localization and functional studies have led to the view that CRN C-termini execute their presumed effector function in the host nucleus, targeting processes required for immunity. These findings have led to great interest in this large protein family and driven the identification of additional CRN-like proteins in other organisms. The identification of CRN proteins and subsequent functional studies have markedly increased the number of candidate CRN protein sequences, expanded the range of phenotypes tentatively associated with function and revealed some of their molecular functions toward virulence. The increased number of characterized CRNs also has presented a set of challenges that may impede significant progress in the future. Here, we summarize our current understanding of the CRNs and re-assess some basic assumptions regarding this protein family. We will discuss the latest findings on CRN biology and highlight exciting new hypotheses that have emanated from the field. Finally, we will discuss new approaches to study CRN functions that would lead to a better understanding of CRN effector biology as well as the processes that lead to host susceptibility and immunity.

## Introduction

Pests and pathogens form some of the greatest threats to global food production, constraining crop productivity in an age that features significant growth of the world’s human population (Oerke, 2006; Newbery et al., 2016). Amongst the biotic threats that wreak havoc on plants destined for consumption, the Oomycota form a distinct lineage of water-dwelling Eukaryotic microbes, many of which form parasitic interactions with plants. Amongst them, members of the *Phytophthora* genus rank amongst the most devastating pathogens, collectively affecting virtually every dicotyledonous crop plant (Lamour et al., 2007; Fawke et al., 2015).

Efforts to mitigate the problems posed by pathogens have included intense research into the processes that specify resistance as well as susceptibility in plants. Genetic, genomic, cell biological and biochemical studies have provided reasonable detail on the plant immune system, its constituent parts as well as the mechanics that prevent plants from succumbing to colonization by a plethora of would-be pathogens (Chisholm et al., 2006; Jones and Dangl, 2006). Besides physical and chemical (constitutive) barriers, plants deploy a set of surface-exposed receptor proteins that are able to bind pathogen-derived, non-self molecules (Pathogen or Microbe-associated Molecular Patterns; P/MAMPs) and initiate Pattern Triggered Immunity (PTI) (Boller and Felix, 2009; Nicaise et al., 2009; Muthamilarasan and Prasad, 2013; Macho and Zipfel, 2014; Bigeard et al., 2015). The ability of plants to detect and respond to a wide range of microbial patterns from their environment, whilst moderating immune responses to levels that allow completion of their lifecycle, is testament to an intricate and finely tuned host immune signaling network. This robust and highly flexible immune system is critical to keep harmful microbes at bay whilst fostering productive plant growth.

Per definition and in a bid to be successful, pathogens must overcome cellular host defenses. This implies that microbes with parasitic lifestyles have acquired and evolved factors that counter immunity associated processes. Indeed, decades of intense research have firmly implicated pathogen-encoded secreted factors (effectors) that suppress immunity and trigger susceptibility in a process dubbed Effector-Triggered Susceptibility (ETS) (Jones and Dangl, 2006; Boller and He, 2009; Oliveira-Garcia and Valent, 2015). Acquisition, evolution, maintenance and expression of large effector repertoires illustrate the importance of perturbing host cellular processes in disease establishment. It is therefore not surprising that host–pathogen arms races have sparked the innovation of accessory systems in plants, able to detect effector activities and mount immune responses. Genome sequencing and functional analyses have unveiled a large and highly diverse receptor protein family in plants (NBS-LRRs) that are widespread across the plant kingdom and enable Effector Triggered Immunity (ETI) (Takken et al., 2006; Lee and Yeom, 2015; Khan et al., 2016). Perhaps not surprisingly, pathogens deploy effectors that either avoid or suppress processes required for ETI and re-establish susceptibility (Block and Alfano, 2011; Oliveira-Garcia and Valent, 2015). With these observations made in numerous host–pathogen systems, a powerful evolutionary model has emerged that explains and to some degree predicts signatures of host–microbe co-evolution (Jones and Dangl, 2006).

Many cellular processes contribute to host immune signaling or PTI (Nicaise et al., 2009; Bigeard et al., 2015). Given that immunity associated processes take place throughout the plant cell, it is perhaps not surprising that effectors fulfil their functions in almost every (sub) cellular host compartment. Localization, functional and biochemical studies have led to the identification of effectors that reside in the host apoplast and act at the extracellular host–microbe interface (apoplastic effectors) as well as pathogen proteins that travel across the host membrane and target intracellular processes (cytoplasmic effectors) (Schornack et al., 2009; Oliva et al., 2010; Giraldo and Valent, 2013; Asai and Shirasu, 2015; Jashni et al., 2015; Lo Presti et al., 2015; Selin et al., 2016). Effector virulence functions have been intensively studied in variety of plant–pathogen systems and combined with genome-wide comparative analyses, have prompted the view that pathogen effectors and their functions are highly diverse, rapidly (co-) evolving (with their host) and often specific to a given pathogen species (Dong et al., 2015).

Oomycete pathogens are notorious agents of disease on crop plants. Studies on the effector biology within this group of organisms have led to the identification of vast effector repertoires, some of which act inside the plant cell (Hein et al., 2009; Schornack et al., 2009). Within the *Phytophthora* genus, two predominant classes of cytoplasmic effectors have been identified and studied, namely the RXLR and CRN effector protein families. Both protein classes feature modular architectures, featuring motifs or domains required for delivery situated at the N-terminus (the RXLR motif for RXLR effectors and the LXLFLAK motif for CRN proteins), followed by C-terminal domains that carry effector functions (Whisson et al., 2007; Schornack et al., 2010). The identification of RXLR proteins within *Phytophthora* and the realization that some members of this family act as avirulence (*Avr*) factors in the presence of specific (intracellular) receptor-like resistance (R-) genes have prompted and driven the discovery of a plethora of effector targets, virulence functions and molecular strategies within this family (Schornack et al., 2009; Bozkurt et al., 2012). These results have led to the view that the RXLR effectors comprise a large repertoire of fast evolving genes, whose products target nearly every subcellular compartment and are confined to a relatively small group of oomycete pathogens (Anderson et al., 2015). The increasing availability of pathogen genomes has not only led to an appreciation of the vast effector arsenals pathogens deploy, but also presented the field with a number of questions, some of which have remained unanswered. One observation for example is that in contrast to the RXLRs, the CRN protein family is widespread across the oomycete lineage (Schornack et al., 2010; Stam et al., 2013b; Zhang et al., 2016). This has raised the possibility that, besides the RXLR protein family, other cytoplasmic effectors, such as the CRNs, exist and have equivalent important roles in triggering host susceptibility. If true, the CRN effector family exemplifies the need to study lesser-known effector classes to fully understand pathogen biology. In this review we will summarize the current state of art on CRN research, explore the biology of these proteins, define open questions and propose ways to improve our knowledge on CRN function toward immunity associated processes in plants.

## CRNs are Part of a Large and Conserved Eukaryotic Protein Family

CRN effectors were first identified in the plant pathogenic oomycete *Phytophthora infestans* where they were found to cause a CRinkling and Necrosis (CRN) phenotype when systemically expressed in plant tissue (Torto et al., 2003). In that study, high throughput cloning was conducted of *P. infestans*-derived cDNA clones, which were identified in an Expressed Sequence Tag (EST) sequencing approach and found to have a predicted signal peptide. Subsequent application of a high-throughput functional expression assay *in planta* led to the identification of proteins that induce cell death upon expression in plants, two of which (CRN1 and CRN2), were found to be related on the sequence level (Torto et al., 2003). Since their discovery in *P. infestans*, equivalent studies in other oomycete pathogens revealed that in contrast to the RXLR protein family, CRN coding genes are widespread in the oomycete lineage. Transcriptome sequencing in the phylogenetically distinct pathogen *Aphanomyces euteiches* for example, also identified CRN effectors, thereby extending their known occurrence beyond the *Phytophthora* genus (Gaulin et al., 2008). These results suggest that CRNs are an ancient class of conserved oomycete effector proteins. Consistent with this finding, subsequent genome analyses have unveiled CRN coding genes in all plant pathogenic oomycetes sequenced to date (Haas et al., 2009; Kemen et al., 2011; Links et al., 2011; Lamour et al., 2012; Adhikari et al., 2013; Stam et al., 2013b; Derevnina et al., 2015; Sharma et al., 2015) although in some genomes, gene family expansion seems to have taken place (Haas et al., 2009; Stam et al., 2013b). Interestingly, CRN-like proteins were also identified in the two basal fungal species *Batrachochytrium dendrobatidis* and *Rhizophagus irregularis*. These results suggest either a horizontal transfer event between organisms or that all these genes were already present in early eukaryote progenitors (Sun et al., 2011; Lin et al., 2014). Regardless of their history, the presence of CRNs in the pathogenic fungus *Batrachochytrium dendrobatidis* and their absence in its closest relative, a non-pathogenic chytrid fungus *Homolaphlyctis polyrhiza*, suggest that these effectors are retained in pathogens and thus form a link with pathogenic processes (Joneson et al., 2011). Recently a comprehensive study employed sequence analysis, structure comparison and comparative genomics to assess CRN occurrence across the Eukaryote taxon (Zhang et al., 2016). This revealed that CRN effectors are not only widespread in parasitic organisms, but also occur in free living eukaryotes and land plants that are not known to have a pathogenic lifestyle, seemingly invalidating the link between CRN presence and pathogenicity (Zhang et al., 2016). It was suggested, however, that CRN like proteins were initially deployed to resolve inter-organismal conflicts, after which in some host-pathogen interactions, these proteins were co-opted as effectors (Zhang et al., 2016).

## CRNs Modular Structure

**FIGURE 1:**
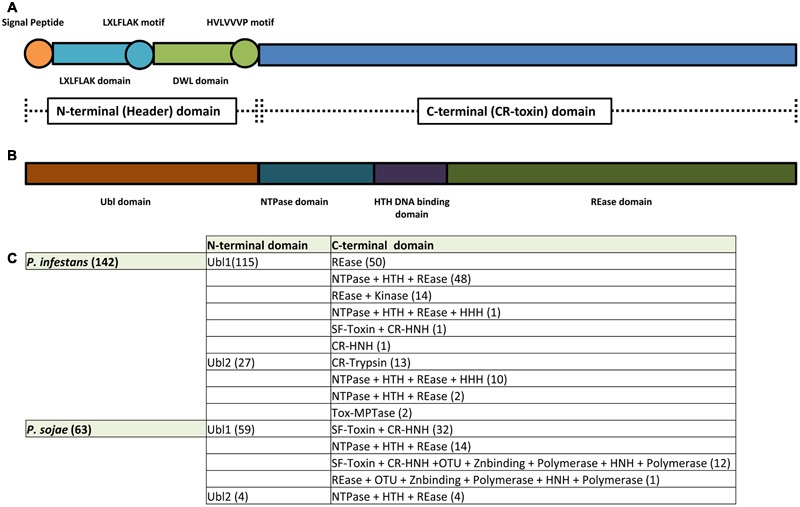
**CR(N) structure analysis.** CRN effectors are modular proteins with an N-terminus thought to be responsible for CRN secretion and translocation into the host and a C-terminus responsible for CRN virulence function(s). **(A)** CRN N-termini were thought to contain a conserved structure featuring: a signal peptide for secretion; an LXLFLAK domain containing the respective LXLFLAK motif connected with translocation; and a DWL domain that ends in a conserved HVLVVVP motif that marks the end of CRN N-terminus and is thought to be a hot spot for recombination events. In contrast, CRN C-termini were shown to exhibit a large variety of domain structures (not depicted here). **(B)** Zhang et al. (2016) redefined CRN structure. CRN N-termini (renamed header domains) from the two *Phytophthora* species analyzed (*P. infestans* and *P. sojae*) all feature an Ubiquitin like (Ubl) domain that is thought to be responsible for secretion and translocation into the host cell. CRN C-termini (also named CR-toxin domains) feature distinct domain architectures, having enzymatic origins. The majority of *Phytophthora* CRN C-termini contained the depicted domain structure (NTPase + HTH + REase). **(C)** Summary of domain architectures predicted to occur in *Phytophthora* (from Zhang et al., 2016). The number of CRN proteins with each given domain architecture/composition are indicated between brackets.

The first conserved regions identified in CRN proteins were found to be situated at the N-terminus, featuring a highly conserved LXLFLAK motif (**Figure 1A**) (Win et al., 2007). Aiming to study the evolution of RXLR effectors, it was observed that 16 *Hyaloperonospora parasitica* effectors showed similarity to CRN proteins, prompting the discovery that the RXLR motif was coupled to the LXLFLAK amino acid sequence (Win et al., 2007). This observation then led to the suggestion that both RXLR and LXFLAK domains are analogous and possibly involved in host targeting. This then implied that CRN proteins are modular with domains that execute distinct functions, i.e., host targeting and signaling perturbation. Subsequent sequence analyses of CRN proteins identified in 3 *Phytophthora* genomes confirmed this notion whilst extending this rule to the entire CRN protein family (Haas et al., 2009). From this it was proposed that the CRNs form a family of modular proteins with a highly conserved N-terminal domain of around 130 amino acids, presumed to specify trafficking and containing both an LXLFLAK motif and diversified DWL domains. In this model, the highly conserved HVLVXXP motif marks the end of the N-terminal region as it is considered a recombination hotspot where C-terminal regions, carrying effector functions are linked up (**Figure 1A**) (Haas et al., 2009). In line with the expectation that effector families and their functions are diverse, subsequent computational analyses on CRN coding genes identified in *P. infestans, P. ramorum*, and *P. sojae* allowed the identification of 36 conserved C-terminal sub-domains. Expression of the C-terminal domains from the previously described CRN2 and four other CRNs led to cell death in *N. benthamiana* plants, suggesting that effector functions are diverse and located at the C-terminus. Given that the N-terminus (and predicted signal peptides) were found to be dispensable for cell death induction and CRN effectors thus seemingly acted inside plant cells, it was suggested that CRN N-termini specify the secretion and translocation of effector domains into the host (Haas et al., 2009).

## CRN Gene Expression and Regulation

To allow successful host colonization, the expression of pathogen genes requires great coordination and thus extensive regulation. This also appears true for effector gene expression, with dynamic and stage-specific changes in effector transcript levels demonstrated repeatedly. Microarray analyses of *P. infestans* mycelia revealed that 98% of all annotated CRNs are expressed and 66% of those were amongst the top 10% when assessed for array signal intensities. These results were similar to those found for RXLR effectors, where 66% of the genes were expressed and 4% were in the top 10% (Haas et al., 2009). Another study showed similar results, indicating that CRNs were expressed to a higher level than RXLR effectors (Shen et al., 2013). Besides high levels of expression, CRN coding genes were also differentially expressed during infection. CRNs from *P. capsici* could be divided in two groups according to their expression patterns. Class 1, forming a group that are upregulated in the early and late stages of infection, while Class 2 CRN gene expression gradually increases to peak in the late infection stages (Stam et al., 2013b).

Whilst CRN gene expression appears to be regulated during the infection process, the principal transcription factors remain to be identified. One possible mechanism of post-transcriptional regulation was unveiled recently in *P. infestans*, when sequencing of small non-coding RNAs led to the discovery of families of sRNAs that were predicted to target CRN coding genes (Vetukuri et al., 2012). Although DCL-like proteins were implicated in the generation of sRNAs by means of gene silencing, the effect of sRNA abolishment on CRN gene expression or pathogen virulence was not assessed (Vetukuri et al., 2012). Further studies will be required to firmly implicate sRNAs in CRN gene regulation in *P. infestans* and other oomycete pathogens. Besides (post) transcriptional regulation, translational control and post-translational modifications form important means by which level of functional effector proteins could be controlled. A quantitative phospho-proteomics study in *P. infestans* revealed that CRN proteins are phosphorylated across distinct life cycle stages (Resjö et al., 2014). Although phosphorylation of CRN8 had previously been demonstrated and implicated in virulence function (van Damme et al., 2012), this study revealed that other CRNs, lacking a kinase domain, are also phosphorylated on residues that are widely conserved within the CRN protein family (Resjö et al., 2014). Whilst phosphorylation of residues was found to be widespread and target conserved domains within the CRN protein family, functional relevance remains to be established. It is likely, however, that use of this and possibly other PTMs, not only help regulate protein function, but also direct events required for secretion, delivery and stability. The study of the kinases responsible as well as their targets will undoubtedly reveal mechanisms required for CRN delivery and function.

## Evidence Supporting Translocation of CRN Proteins into Host Cells

Per definition, cytoplasmic effectors need to reach the cell interior to function toward their host target(s). Whilst computational and deletion analyses pointed at a role for CRN C-terminal domains inside the host cell and implicated N-termini in delivery, more concrete evidence emerged from functional studies in *Phytophthora capsici*. Using a *Phytophthora* transformation approach, constructs carrying the *P. infestans* Avr3a coding gene were first introduced in *P. capsici* and resulting strains used to infect transgenic *N. benthamiana* leaves expressing the potato resistance protein R3a. Whilst expression of AVR3a led to avirulence in these assays, strains that expressed AVR3a versions with a mutated RXLR motif, remained virulent, mirroring results in *P. infestans* (Whisson et al., 2007) and suggesting that AVR3a translocation conditions avirulence in the *P. capsici*-*N. benthamiana* system (Schornack et al., 2010). The ability of R3a to detect translocation in these assays was then used to show that the N-termini of CRN2, CRN8 and CRN16 mediate host-trafficking of the AVR3a C-terminus, evidenced by avirulent outcomes in infection assays on R3a expressing leaves, but not in the absence of R3a (Schornack et al., 2010). Importantly, equivalent experiments using *P. capsici* strains expressing CRN-AVR3a fusion proteins in which the N-terminal LFLAK motif was mutated to LAAAA, led to infection. These results suggested that the LXLFLAK motif helps CRN trafficking into the host cell and provided a rationale for the use of CRN N-terminal sequences for genome wide searches, aimed at identifying and cataloging candidate CRN effectors in pathogenic oomycete genomes.

A recent study shed a different light onto the supposed requirement of LXLFLAK motifs in CRN translocation (Zhang et al., 2016). Genome surveys spanning the eukaryote taxon uncovered CRN N-termini that lacked the LXLFLAK motif. Moreover, those that contained this motif were predicted to have an ubiquitin-like structure, similar to those found in the N-terminal region of SSK1/Mcs4 signaling proteins in fungi. In these analyses, the LXLFLAK motif was located in strand 2 and 3 of this ubiquitin-like domain, suggesting that structural features rather than sequence conservation underpin CRN translocation (**Figure 1B**) (Zhang et al., 2016). With a great number of “atypical” CRN N-termini identified, their contribution to translocation activity requires testing *in vivo*.

## CRNs Target Host Nuclear Processes

In contrast to the RXLR effector class, all CRN effectors localized to date accumulate in the nucleus when expressed *in planta* (Stam et al., 2013b). As one would expect, nuclear localization was found to be required for effector function in a number of cases, supporting the idea that CRN proteins target host nuclear processes. For example, the *P. infestans* CRN8 protein localizes to the nucleus and causes cell death, a phenotypic outcome thought to reflect virulence function (van Damme et al., 2012). Silencing of importin-α, a component of the nuclear pore complex required for active transport of proteins into the nucleus, led to altered PiCRN8 localization and a reduction in cell death (Schornack et al., 2010). In addition, fusion of a nuclear exclusion signal (NES) to this effector drastically impeded nuclear accumulation and cell death occurrence (Schornack et al., 2010), supporting the idea of nuclear localization requirements. Similar results were obtained for *P. sojae* and *P. capsici* CRNs PsCRN63 and PcCRN4 (also known as PcCRN83_152) respectively (Liu et al., 2011; Mafurah et al., 2015). However, PsCRN115, a CRN highly similar to PsCRN63 but without cell death inducing capacity, was shown to be able to supress cell death processes even when its nuclear localization signal was mutated (Liu et al., 2011). Thus, while it looks like nuclear localization is required for CRN cell death activity it remains unclear if suppression of plant defenses requires accumulation in the nuclear compartment.

## Unveiling CRN Virulence Functions

The fact that all CRN effectors accumulate in the host nucleus could indicate that they are targeting identical or a limited set of host processes. By extending the link between localization and function, however, this hypothesis is improbable as CRNs show different sub-nuclear localization patterns (Stam et al., 2013a,b). In addition, more detailed functional analyses have highlighted distinct cell death induction profiles and differential effects on PTI (Stam et al., 2013a), all supportive of diverse functions within this family. This observation is supported by recent work, aimed at understanding the virulence targets and functions for a growing set of CRN proteins (Stam et al., 2013c; Ramirez-Garcés et al., 2015; Song et al., 2015; Zhang et al., 2015b).

Further studies have reinforced the notion of functional diversity whilst revealing phenomena that remain unexplained. Transient expression of two CRN effector domains in *N. benthamiana*, differing by only 4 amino acids, revealed opposing functions (Liu et al., 2011). PsCRN63 induced necrosis in plants while PsCRN115 was found to suppress cell death (Liu et al., 2011). Furthermore, over-expression of PsCRN115 was shown to enhance plant immunity whilst for PsCRN63 a decrease in resistance was observed (Zhang et al., 2015a; Li et al., 2016). Interestingly, both effectors were shown to directly interact with plant catalases and interfere with hydrogen peroxide (H_2_O_2_) accumulation. PsCRN63 was shown to increase H_2_O_2_ accumulation while PsCRN115 was shown to suppress this process. It was also suggested that PsCRN63 recruits plant catalases into the host nucleus leading to catalase destabilization while PsCRN115 inhibits these events (Zhang et al., 2015b). Consistent with a role in infection, simultaneous silencing of both genes led to a reduced virulence phenotype on soybean (Liu et al., 2011), supporting the idea that these proteins are bona fide effectors. However, given that both genes were silenced, it remains unknown to what extent each effector contributes to virulence. Taken together, these results, though perhaps counterintuitive, provide some insights into the means by which this effector pair exhorts its function in plants. Nevertheless, whether these observations are extendible to the CRN protein family as a whole or if other CRN effector pairs exist, remains to be seen.

Importantly, despite being named after their ability to cause crinkling and cell death, cell death inducing activity is not a characteristic common to all CRN effectors. Moreover, it appears that many CRNs that do not induce necrosis, supress host cell death processes. For instance, Shen et al. (2013) selected 10 *P. sojae* CRN effectors and tested for their cell death inducing and suppression capacities respectively. Only one of these CRNs (PsCRN172-2) induced cell death when over-expressed in *N. benthamiana* leaves. The remaining 9 were able to suppress cell death caused by PsojNIP; 8 by PsCRN63; 5 by Avr3a + R3a; and 3 by Avh241 (Shen et al., 2013). Thus, it seems more likely that CRN effectors act as cell death regulators in host plants rather than inducers. However, in the absence of concrete evidence connecting cell death induction to virulence function, the significance of CRN induced necrosis remains a matter of speculation.

Interestingly, PsCRN63 was suggested to form homo-dimers and this dimerization was shown to be required for its ability to supress plant immunity processes and mediate host cell death (Li et al., 2016). Moreover, the authors suggested that PsCRN63 was able to form dimers with PsCRN115 and with unrelated PsCRN79 and PcCRN4. Thus, there is a possibility that PsCRN115 is increasing plant immunity by repressing PcCRN63 cell death in a dominant-negative manner. In addition, these authors suggest that the dimerization process could be widespread in CRN effectors, leading to the hypothesis that CRNs form complexes to enhance pathogen virulence (Li et al., 2016). If true, this hypothesis opens exciting research opportunities. However, it also raises new challenges on designing experiments and on drawing significant conclusions when studying individual CRN functions. In another major advance, it was demonstrated that the C-terminal half of PiCRN8 from *P. infestans* has kinase activity and is auto-phosphorylated when expressed in plant cells. In this work a kinase dead mutant of PiCRN8 was generated and was shown to have dominant-negative effects on PiCRN8 cell death and to reduce *P. infestans* virulence when over-expressed *in planta*. Interestingly, and based on this work, PiCRN8 was also suggested to dimerize *in planta* (van Damme et al., 2012).

## CRNs Bind and Modify Host Targets to Promote Virulence

Besides the interaction of PsCRN115 and PsCRN63 with plant catalases (Zhang et al., 2015b), there are few other examples of identified CRN host targets. A matrix yeast two hybrid screen identified *Arabidopsis* TCP14 as a major hub targeted by *Hyaloperonospora arabidopsidis* and *Pseudomonas syringae* effectors, including 3 *H. arabidopsidis* CRN effectors (Arabidopsis Interactome Mapping Consortium, 2011; Mukhtar et al., 2011). Over-expression of a TCP14 from tomato, SlTCP14-2, was shown to enhance immunity against *P. capsici*. *P. capsici* CRN, CRN12_997, was shown to directly bind SlTCP14-2, abolishing the immunity increase mediated by SlTCP14-2. CRN12-997 is proposed to achieve this immunity increase abolishment by diminishing SlTCP14-2 association with DNA and by modifying SlTCP14-2 sub-nuclear localization (Stam et al., 2013c).

More recently two CRN effectors were shown to achieve their virulence functions by interacting with host DNA. *P. sojae* PsCRN108 was shown to contain a putative DNA-binding helix-hairpin-helix (HhH) motif that inhibits the expression of Heat Shock protein (HSP) genes in *A. thaliana, N. benthamiana* and soybean. This is achieved by the binding of PsCRN108 to conserved promotor regions of HSP genes named heat shock elements (HSEs). HSEs are bound by heat shock transcription factors (Hsf’s) leading to tight regulation of HSP expression. PsCRN108 was shown to be able to inhibit the binding of the Hsf AtHsfA1a which induces HSP gene expression in response to stress (Song et al., 2015). Another study aimed to investigate the function of two related CRNs from the plant pathogenic oomycete *Aphanomyces euteiches* (AeCRN13) and from the amphibian pathogenic chytrid fungus *Batrachochytrium dendrobatidis* (BdCRN13) also showed that both these effectors directly interact with DNA. These two cell death inducing effectors contain an HNH-like endonuclease motif that triggers plant DNA damage response (DDR). Mutation of key residues in the AeCRN13 HNH-like endonuclease motif abolished AeCRN13 capacity to interact with DNA, to induce DDR and to increase the susceptibility of *Nicotiana Benthamiana* to *P. capsici*. Thus the function of the HNH-like endonuclease motif on inducing DDR has been connected to AeCRN13 virulence function (Ramirez-Garcés et al., 2015).

## From Transposons to Toxins: A Role for CRN Proteins in Inter-Organismal Conflicts?

Recently a comprehensive study employed a combination of sequence analysis, structure prediction and comparison as well as comparative genomics to assess CRN occurrence across the Eukaryote taxon (Zhang et al., 2016). This study revealed that CRN effectors are not only widespread in parasitic organisms, but also occur in free living eukaryotes and land plants that are not known to have a pathogenic lifestyle (Zhang et al., 2016). The identification of CRN proteins in such a variety of organisms lead to their association with previously described proteins. Predicted proteins that resemble CRNs were found in trypanosomes where they are regarded as Retrotransposon Hot Spot Proteins (RHSPs). RHSPs are expressed in the vicinity of genes required for pathogenesis and immune-invasion (Bringaud et al., 2002; Zhang et al., 2016). The association of CRNs with RHSPs lead authors to rename CRN proteins into CR (Crinkler-RHS-type) proteins (Zhang et al., 2016).

Making use of a vast collection of CR-proteins, Zhang et al. (2016) analyzed and characterized CR domain structure by searching extensive databases of sequence profiles, including PFAM. Then CR proteins were compared to identify and delineate conserved domains that could be used for classification (using tools as PSIBLAST; HMM; JACKHMMER; and HHpred). Using this approach, a novel and comprehensive characterization of CRN domain architecture was achieved (**Figure 1B**) (Zhang et al., 2016). In this analysis, the conventional division of CRN proteins into N-terminal domain (thought to be responsible for effector translocation) and C-terminal domains (though to be responsible for virulence effects) (Haas et al., 2009) remains unchanged. However, our views on CRN domains are greatly challenged by this analysis and important possible insights gained on evolution and function. Firstly, the authors ruled out the presence of signal peptides, thought to be present at CRN N-termini. These N-terminal regions, defined as header domains, were predicted to form a ubiquitin-like (Ubl) fold in which predicted signal peptides as well as the conserved LXLFLAK motif are situated at conserved strands-1 and -3 respectively (**Figure 1B**). From this, the authors suggest that the LXLFLAK motif is important for translocation as they are important for Ubl domain structure. This Ubl N-terminal domain is significantly related to those found in fungal signaling proteins, namely SSK1/Mcs4. SSK1 orthologs play important roles in stress responses in various true fungi, and in some cases, are known to do so in a phosphorylation-dependent manner, employing an interaction between their N-terminal domains and a MAPKKK heteromer (Calera and Calderone, 1999; Calera et al., 2000; Chauhan et al., 2006; Morigasaki and Shiozaki, 2013; Yu et al., 2016). From this, the authors suggest that CRN Ubl N-terminal domains could facilitate translocation inside the host and/or the host nucleus by analogous mechanisms (Zhang et al., 2016). To what extend these hypotheses ring true in oomycete-host interactions, however, remains to be determined *in vivo*.

Besides the Ubl domain, CR N-termini feature various unrelated alpha-helical domains, somewhat conserved in a diverse set of organisms (Zhang et al., 2016). Header domains thus appear structurally distinct, suggesting a variety of mechanisms that govern translocation into the target cell. Despite this assumption, only one N-terminal domain, from *Angomonas*, shows a hydrophobic region implying possible membrane interactions and secretion. Moreover, CR proteins from diverse eudicot plants contain CR headers that contain helix-turn-helix (HTH) domains also found in the Myb transcription factor family. In Myb transcription factors, these domains are implicated in DNA-binding, suggesting that these eudicot proteins might not be secreted but target intracellular invasive DNA (Zhang et al., 2016).

**FIGURE 2:**
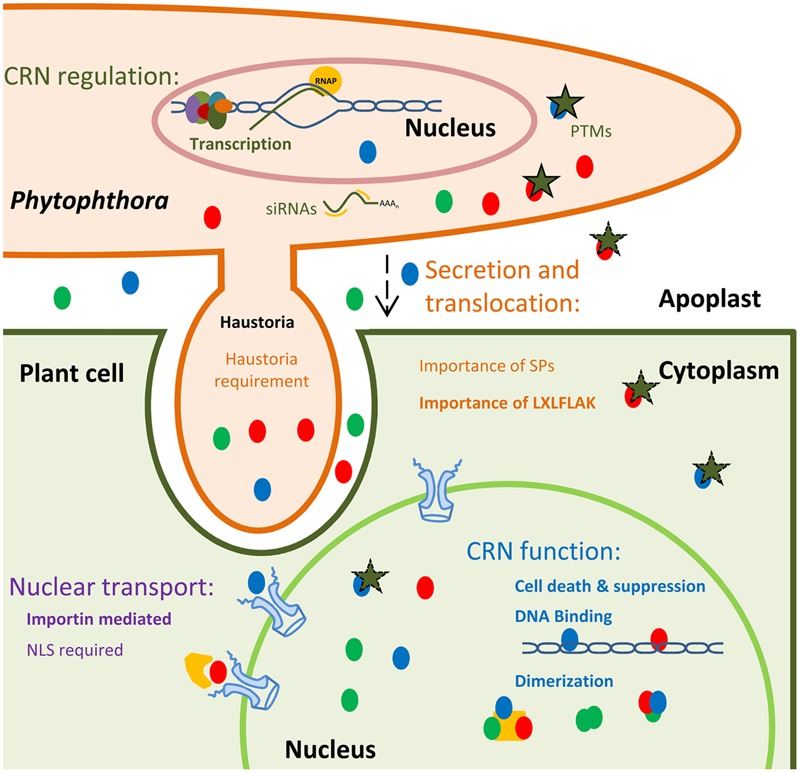
**Schematic representation of current knowns and unknowns in CRN effector biology.** CRNs (depicted as colored circles) are highly expressed and regulated during infection, suggesting transcriptional control. It has been suggested that CRN could be regulated via siRNAs and PTMs, namely phosphorylation (green stars). A wide variety of CRNs have been identified as being phosphorylated in pathogen structures. However, the post-translational status inside plant cells or the apoplast (during transit) remains unknown. CRN secretion and translocation mechanisms remain widely uncharacterized. CRN N-termini were shown to be sufficient to mediate protein secretion and translocation into plant cells. The presence of the LXLFLAK motif was also shown to be required for this process. However, if CRN translocation is achieved in haustoria or if CRN predicted signal peptides are functional remains unclear. CRNs target host nuclear processes, but the mechanisms of trafficking into the nucleus, remain unknown. Importins mediate nuclear import by binding Nuclear Localization signals (NLSs), present in most proteins destined for the nucleus. However, import of CRNs without predicted NLSs has been observed. CRNs have been shown to mediate or suppress cell death processes. Besides proteinaceous nuclear host targets, CRNs have also been shown to target host DNA. Diverse CRNs were shown to form complexes in plant tissues. However, the nature of these dimers with regards to exact composition remains unclear.

As for CRN N-terminal domains, re-classification of CR C-termini have afforded new insights into CR(N) biology. In contrast to CRN N-termini and consistent with previous observations, C-terminal domains are highly diverse and often resemble enzymes (**Figure 1C**). Although high levels of diversity are known to be present, classification led to a limited set of domain configurations that were found to be prevalent. For example, CR C-termini containing a P-loop NTPase domain, combined with a nuclease domain of the restriction endonuclease (REase) superfamily were found to account for slightly more than one-fourth of all CR C-termini. In addition, CR C-termini in which a REase superfamily domain is coupled to protein kinase domain was found to account for approximately one-sixth of the C-termini domains present in the dataset. In both cases, the toxicity function is believed to be specified by the REase domain, whilst the NTPase and Kinase domains would regulate REase activity or affinity toward nucleic acids (such as DNA) (Zhang et al., 2016). This view complies with studies on CRN8 in which disruption of kinase function did not abolish CRN cell death, but mutations in the newly annotated REase domain did (van Damme et al., 2012; Zhang et al., 2016). Moreover, these results indicate that targeting of nucleic acids such as DNA could be a defining feature, shared amongst CRN proteins (**Figure 2**). Indeed, this model is consistent with exclusive localization of CRN proteins to the nucleus and importantly, two recent reports demonstrating binding of CRN effectors to DNA (Ramirez-Garcés et al., 2015; Song et al., 2015). Several other domains were identified as present in CR C-termini, including DNA binding domains (HNH nuclease and LK-nuclease), peptidase domains (trypsin, zincin-like metallopeptidase, and Ulp1- like peptidase), GTPase domains and non-enzymatic or transposon derived domains (Zhang et al., 2016) (**Figure 1C**). Thus, the prediction of enzymatic domains in CR or CRN proteins represents one important mean by which new hypotheses about CRN function can be constructed and subsequently tested (Zhang et al., 2016).

The structural analysis of CR proteins also unveiled similarities to proteins found in prokaryotes, allowing us to infer the evolutionary origin of CR proteins. NTPease coupled with REase domains are widespread in prokaryotes and linked with transposable elements. The role of transposable elements in the regulation of gene transcription and regulation as well as chromatin structure has been well established and therefore these elements are considered motors that drive genome plasticity and adaptation in all kingdoms of life (Hua-Van et al., 2011). Consistent with this view, *P. infestans* CRN coding gene PITG_23144 was shown to have a gypsy retrotransposon inserted in its C-terminal domain (Haas et al., 2009). Even more striking was the discovery that *P. infestans* CRN coding genes, carrying the DC domain, are concentrated in genomic regions enriched for helitron transposons. Moreover, several CRN copies were found in a perfect tail-to-head conformation, mirroring arrangements seen for helitrons throughout the *P. infestans* genome (Haas et al., 2009). Given that helitrons are considered important factors that mediate gene duplication, exon shuﬄing and genome evolution (Kapitonov and Jurka, 2007), one hypothesis that has emerged is that CRN recombination and evolution is helitron mediated.

In contrast to CR C-terminal domains, there is no evidence for the presence of CR N-terminal domains in prokaryotes. CR C-terminal domains are therefore believed to have originated from prokaryotic proteins. This observation and the apparent activities of CR proteins toward nucleic acids, have led to the suggestion that CR-proteins originally evolved in prokaryotes in response to invasive intracellular DNA. Multiple lateral gene transfer events and subsequent coupling of CR proteins to a variety of header domains, allowed these toxins to be co-opted as effector proteins in eukaryotes. The observations and hypotheses emanating from work summarized here, provide a conceptual framework that in turn should lead to new experimental studies that inform on the biology of this ancient protein family in a range of eukaryote organisms (Zhang et al., 2016).

## New Approaches to Study CRN Biology and Functions

With the increasing availability of pathogen genomes, understanding effector mode of action remains a major challenge and bottleneck. Despite recent efforts, new and more systematic ways are required to further understand CRN effector biology. Here we describe the areas where our knowledge on CRN effector biology remains poor or new opportunities have arisen for further exploration (summarized in **Figure 2**). Furthermore, we suggest new ways of tackling these areas, by taking advantage of our knowledge on CRN domain structures, plant–pathogen interactions and effector classes that are better understood.

The mechanisms required for CRN secretion and translocation into the host cell remain largely uncharacterized, due to the absence of tools that allow a comprehensive study on the translocation process. Whilst RXLRs are believed to be translocated in haustoria (Anderson et al., 2015), CRN proteins appear to be present in pathogens that do not form haustoria, leading to the hypothesis that they might use distinct translocation mechanisms (Schornack et al., 2010; Zhang et al., 2016), (**Figure 2**). One way of confirm this notion is to create *Phytophthora* strains, unable to form haustoria, by disrupting factors required for their formation, such as Haustorial Membrane Protein 1 (HMP1) (Avrova et al., 2008). The successful application of CRISPR/CAS9 mediated gene editing in *Phytophthora* should allow the creation of such strains, provided they infect host plants to some degree, which in turn can be used for AVR3a based translocation assays on R3a plants. If feasible, this would tell us if CRNs require haustoria for their delivery and in addition, allow critical analogous experiments for the RXLR effector class. To gain further independent insights into translocation requirements, the identification and study of pathogen and host factors, able to interact with CRN N-terminal or CR-header domains would be of extreme use. Now that predicted structures for CR-header domains are available, rationalization of candidate interactors in the context of translocation mode of action is ever more plausible.

CRN N-termini and CR-header domains have been divided into a diverse set of sub families raising the possibility that not all CR or CRN N-termini facilitate translocation (Zhang et al., 2016). To help resolve this important and biologically interesting observation, translocation experiments should be conducted using representatives of these different families. In such experiments, the presence or absence of predictable signal peptides should be taken into consideration as this may lead to discovery of new and unconventional secretion pathways or refinement of prediction software already available. Taken together, this information may unveil distinct translocation and regulatory mechanisms, governing protein trafficking in diverse eukaryote systems.

As with the N-terminal domains, CRN C-terminal structure could be used to help us hypothesize on CRN function as proposed by Zhang et al. (2016). However, available experimental data demands some caution. In *P. infestans*, CRNs that share predicted effector (sub) domains, feature contrasting cell death inducing activities (Haas et al., 2009). Even more striking are the cases from *P. sojae* where only 7 amino acid differences between PsCRN172-2 and PsCRN172-1 specify cell death inducing and suppressing activity respectively (Shen et al., 2013). Whilst these results mirror the PsCRN63 and PsCRN115 scenario, in which effectors only differ in 4 amino acids, exploration in other *Phytophthora* spp. will help determine whether these finding describe a general rule. Given that predicted structures now are available for CR and CRN proteins, mechanistic studies that aim to unravel the means by which CRN activity is regulated inside the host cell, will be of great value in our efforts to rationalize effector sequence-to-function relationships.

Despite the need for caution when over-interpreting sequence similarity, it would be of extreme value to be able to recognize which domains are present in each CRN, allowing inter-species and inter-article comparisons. For this we believe that it would be of extreme use to the field to agree on a CRN naming convention containing reference to the CRN N-terminal and C-terminal domain structure. The classification presented by Zhang et al. (2016) should be of use, especially when more structural data will be available in the future, allowing further refinement of sub family descriptions.

We already addressed the importance of clarifying the mechanisms used by CRN effectors to achieve translocation into the host cell. As all CRNs localize to the nuclear compartment, it would be interesting to understand the mechanisms used by CRNs to achieve nuclear translocation. It was shown that CRN nuclear localization was mediated by the host machinery, namely by importin-α, as a cytoplasmic localization shift of CRN C-terminal domains fused to GFP was observed in *N. benthamiana* plants silenced for importin-α homologs *NbImpα1* and *NbImpα2* (Schornack et al., 2010). Since importin-α has been shown to mediate nuclear import by binding nuclear localization signals (NLS) in its cargo-substrates (Christie et al., 2015), it is not surprising that CRN proteins that carry NLS signals, travel to the nucleus in an importin-α dependent manner. Intriguingly, CRN proteins that lack a predictable NLS also can accumulate in the same way, suggesting that alternative mechanisms are at play (i.e., bound to another nuclear protein) or that the NLS prediction algorithms are not accurate, generating false negative results. Importin-α has been shown to interact with atypical NLS (Christie et al., 2015), so it is possible that the presence of a predictable NLS is not a strict requirement for transport to the host nucleus. The observation that CRN proteins can form dimers in plant cells, opens up the possibility of effector co-operation in trafficking.

Another significant question that remains unanswered in the field is the importance of CRN mediated cell death and its relevance to virulence. Cell death and virulence phenotypes coincide in several cases (Liu et al., 2011; Stam et al., 2013b; Song et al., 2015) suggesting that cell death represents a phenotype desired by the pathogen. An alternative view, however, is that cell death is an artifact associated with over-expression of an effector function. The observation that a small number of amino acid changes turn a cell death inducing protein into a cell death suppressor suggests that cell death inducing activity may not be a critical function driving infection in the host. Furthermore, with many CRN proteins not inducing any cell death, it certainly is not a defining feature of this protein family. On the other hand, the suggestion that CR(N) proteins may have had toxin functions in a distant past would argue otherwise. For this reason, it would be of great interest to characterize the mechanisms underlying CRN mediated cell death and their connection with virulence activity. Given that some CRNs were shown to induce cell death at different rates (Stam et al., 2013a), it will be important to assess the levels of protein expression and experimental procedures to enable comparisons between cell death and non-cell death inducers.

With CRN effectors being highly expressed and having the ability to cause host cell death, it seems it would be necessary for CRNs to be tightly regulated during the infection process. As discussed above, post-transcriptional and post-translational control could be associated with this regulation. A better understanding on the control of CRN activity on both the transcript and protein level should allow key insights into effector as well as pathogen biology. As stated above siRNAs and PTMs, namely phosphorylation, could be responsible for CRN regulation. However, these two processes have not been connected with the control of CRN function to date. Understanding Post-translational modifications, in particular those that occur in *Phytophthora* and may not take place upon over-expression in plants, could help further (re)define the (cell death) activities of this protein family in more detail.

An important factor, complicating the interpretation of cell death or virulence phenotypes is the apparent ability of CRN proteins to form homo-dimers or dimerize with other CRN effectors (Li et al., 2016). While it is not clear whether host proteins are part of these complexes, it is likely that diverse CRN effector complexes could modify host targets in distinct ways. This raises new challenges in experimental design, as a number of CRN effectors may be unable to achieve their true virulence functions alone. Although CRN co-expression could be attempted, the number of CRN combinations would render these experiments unfeasible, although a set of sensible criteria (gene expression, virulence functions, etc.) could be implemented in a bid to reduce complexity. Systematic Yeast two Hybrid analyses or screens *in planta* should further rationalize intense future studies on CRN effector complex function in host-microbe systems.

Despite the valuable efforts aiming for the identification and characterization of CRN virulence functions, CRN effector biology remains still largely uncharacterized. However, with our available knowledge on CRN distribution and structure, and with the ever improving techniques that enable an efficient study of plant-pathogen interactions we are on the verge of truly unveiling the role of CRN effectors and their biology. Indeed, CRN effector biology is emerging as a fertile research area where new and possibly game-changing concepts in effector biology may be discovered.

## Author Contributions

TA and EH wrote the paper. GT and GM contributed with comments and suggestions.

## Conflict of Interest Statement

The authors declare that the research was conducted in the absence of any commercial or financial relationships that could be construed as a potential conflict of interest.
